# Total atrial conduction time provides novel information in prediction for stroke in patients with sinus rhythm

**DOI:** 10.1007/s00380-022-02189-7

**Published:** 2022-10-20

**Authors:** A. G. Bejinariu, M. Schilling, P. Müller, L. Clasen, S. Gerguri, S. Angendohr, S. Katsianos, J. Schmidt, C. Brinkmeyer, S. G. Meuth, M. Kelm, H. Makimoto

**Affiliations:** 1 Division of Cardiology, Department of Cardiology, Pulmonology and Vascular Medicine, Medical Faculty, Heinrich-Heine University, Moorenstrasse 5, 40225 Düsseldorf, Germany; 2 Division of Neurology, Medical Faculty, Heinrich-Heine University, Moorenstrasse 5, 40225 Düsseldorf, Germany; 3 CARID, Cardiovascular Research Institute Düsseldorf, Medical Faculty, Heinrich-Heine-University, Moorenstrasse 5, 40225 Düsseldorf, Germany

**Keywords:** Stroke, Atrial fibrillation, Echocardiography

## Abstract

The total atrial conduction time (TACT) measured by echocardiography predicts the risk of atrial fibrillation (AF). This study aimed to investigate whether adding the TACT to the revised Framingham stroke risk profile (rFSRP) improves the efficacy of predicting stroke incidence in patients without prior stroke or known AF. The TACT was measured in 376 consecutive patients > 18 years (58.5 ± 16.3 years; 46% male) receiving echocardiography without any prior history of stroke or AF. The primary endpoint was the occurrence of ischemic stroke, and the secondary endpoint was any documentation of AF during the 2 years of follow-up. During the follow-up period, ischemic strokes occurred in 10 patients (2.65%), and AF in 22 patients (5.85%). The TACT was significantly longer in those who later had a stroke compared with those who did not (169.4 vs. 142.7 ms, *p* < 0.001). Both rFSRP and TACT predicted the risk for stroke incidence. The univariate model showed that the TACT was a predictor of ischemic stroke incidence (*p* < 0.001; hazard ratio of 1.94 for every 10 ms; 95% confidence interval, 1.49–2.54). The addition of TACT to rFSRP significantly improved the area under the receiver operating characteristic curve (0.79 vs. 0.85, *p* = 0.001). Stroke risk prediction was significantly improved by the addition of TACT to rFSRP. The utility of the TACT should be further investigated in large-scale randomized clinical trials.

**Supplementary Information:**

The online version contains supplementary material available at 10.1007/s00380-022-02189-7.

## Introduction

Stroke is, according to the World Health Organization, the second leading cause of death worldwide [1, 2]. In 2017, 8% of the deaths in Europe were attributed to acute stroke. Moreover, stroke survivors often suffer from severe disability, which in turn generates an elevated use of health and social care resources, up to 8% [2]. Improving the current methods of stroke risk stratification may aid clinical decision-making when considering stroke prevention therapies, thereby reducing the burden of stroke on patients and resources.

Atrial cardiopathy has recently emerged as a significant precursor for stroke, which has led to the suggestion of a new model for the mechanism of stroke pathogenesis in atrial fibrillation (AF): aging and systemic vascular risk factors cause abnormal atrial tissue substrate (atrial cardiopathy), which results in both AF and thromboembolism [3]. Of note, it is well established that the risk of stroke is strongly associated with AF [4].

The total atrial conduction time (TACT), measured noninvasively as PA-TDI interval (the time interval from initiation of the P wave to the peak of the A’ wave of the atrial tissue Doppler tracing) [5, 6], has been shown to be correlated with the degree of atrial fibrosis and predict the occurrence of postoperative AF after cardiac surgery[5] as well as predict stroke risk in patients with AF, independent of the CHA_2_DS_2_-VASc score [6].

In the general population, stroke risk can be calculated using the revised Framingham stroke risk profile (rFSRP), which has been recently described and validated in two external cohorts (including patients without a history of AF) [7]. However, the rFSRP may not directly reflect atrial cardiopathy, as this scoring system does not include any echocardiographic or electrocardiographic parameters [8–10].

Therefore, we hypothesized that the TACT, as a surrogate parameter for atrial cardiopathy, improves the efficacy of stroke prediction when added to the rFSRP in patients without known AF.

## Materials and methods

### Study ethics

The study protocol was approved by the local ethics committee of Heinrich Heine University Düsseldorf (Study Number 5228R). Informed written consent was obtained from all study participants.

### Study population

We prospectively enrolled consecutive patients in our echocardiography laboratory. Patients were considered eligible if they underwent transthoracic echocardiography, were older than 18 years of age, and provided informed consent before inclusion. Patients were excluded if they had a history of stroke (ischemic or hemorrhagic) or AF/atrial flutter (at least one episode lasting longer than 30 s). Patients with a history of hospital admission due to decompensated heart failure within 6 months of the date of recruitment were also excluded.

Comorbidities, medications, and epidemiological data were recorded. In addition to the echocardiographic assessment, we calculated the rFSRP to determine stroke risk at 2 years [7]. The follow-up period was planned to be 24 months.

### Echocardiography

A Vivid 7 machine (GE Healthcare, Chicago, IL, USA) was used to perform echocardiography. Two-dimensional transthoracic echocardiography was performed in all patients, and echocardiographic parameters were assessed according to the guidelines of the American Society of Echocardiography and were supervised by experienced cardiologists certified in adult transthoracic echocardiography by the European Association of Cardiovascular Imaging [11]. Essentially, left atrial (LA) diameter was measured at the end-systolic time point in the parasternal long axis view, and the left ventricular ejection fraction was measured using the bi-plane method of disks (modified Simpson’s rule, apical four-chamber and two-chamber views, respectively).

The TACT was estimated using the PA-TDI interval. This was measured twice for every patient, and the mean value was then calculated. In the apical four-chamber view, the pulsed-wave tissue Doppler sample was placed on the lateral wall of the LA above the mitral annulus. The time interval from initiation of the P wave (lead II as recorded by the echocardiography machine) to the peak of the A’ wave of the atrial tissue Doppler tracing, as shown in Online Resource 1, was defined as the PA-TDI interval, as previously described [12].

### Follow-up

The primary outcome was defined as the occurrence of ischemic stroke (both major and minor [13]) during the 2 years of follow-up. The secondary outcome was defined as newly diagnosed AF (at least one episode lasting longer than 30 s) within 2 years.

Major stroke was defined as an episode of neurological dysfunction caused by focal cerebral, spinal, or retinal infarction, with symptoms persisting for longer than 24 h [14]. Minor stroke was diagnosed in patients with a National Institutes of Health Stroke Scale score of ≤ 3 points. AF was diagnosed by 12-lead electrocardiogram (ECG) and Holter monitor.

A detailed overview of the inclusion process and follow-up is presented in Online Resource 2 (panel A).

The necessary sample size was calculated prior to patient recruitment. Specifically, we hypothesized that patients with prolonged TACT will have a higher incidence of stroke than patients without TACT prolongation during the 2 years of follow-up. We estimated the annual incidence of patients with and without TACT prolongation, and without known AF, as 2.5% and 0.25%, respectively, based on previous work from other groups [6, 15, 16]. For a 2 years follow-up period, with a dropout rate of 15% and one-tailed testing, a total of 367 participants were calculated to be necessary (statistical power of 0.8).

### Statistics

Data were analyzed using SPSS statistical software version 23 (SPSS Inc., Chicago, IL, USA). Continuous variables are expressed as mean ± standard deviation (or median and interquartile range [IQR]), and categorical parameters are expressed as counts and percentages. Normal distribution was assessed using the Shapiro–Wilk test. Study participants were divided by outcome, primarily whether they had suffered a stroke in the follow-up period of 24 months. We compared the demographics, clinical characteristics, and echocardiography data of both groups using the *t* test for continuous variables and Fisher’s exact test for categorical variables. Univariate and multivariate Cox regression analyses were performed to assess predictive values for ischemic stroke. Receiver operating characteristic (ROC) curve analysis was performed for rFSRP, TACT (via the PA-TDI method) alone and in combination, as well as for the CHA_2_DS_2_-VASc score. In order to achieve this, we combined the rFSRP and TACT into a single composite score using logistic regression model and evaluate the predictability of markers by the one-dimensional composite score [17]. Statistical significance was set at *p* < 0.05.

## Results

### Patients included

Based on the power calculation, we screened 408 patients with informed consent obtained from 393 patients. As shown in Online Resource 2 (panel A), follow-up was completed in 376 patients. We included only 376 patients who completed the follow-up in our analysis and the subsequent description of the results. Seventeen patients who were lost to follow-up (4.3%) demonstrated no significant differences in terms of clinical characteristics compared with those with complete follow-up. Baseline clinical characteristics are summarized in Online Resource 3. The mean age of our cohort was 58.5 years (median, 61 years; IQR, 23), and 54% (*n* = 203) were male.

### Cardiovascular events during follow-up

During the follow-up period of 2 years, 10 patients (2.6%) experienced an event: 6 minor strokes and 4 major strokes, all of which were ischemic. Twenty-two patients were newly diagnosed with AF. Of note, both a diagnosis of stroke and new AF were noted in two patients.

The clinical characteristics of the patients without subsequent stroke (*n* = 366 [97.4%]) compared to those with stroke (*n* = 10 [2.6%]) demonstrated significant differences with regard to age (61 vs. 75, *p* = 0.003), peripheral arterial disease (10.9 vs. 40%, *p* = 0.005), beta-blocker medication use (36.9 vs. 70%, *p* = 0.03), and virtual CHA_2_DS_2_-VASc score (Table 1). The rFSRP at 2 years was significantly higher in patients with subsequent strokes than in those without (1.93 vs. 0.33%, *p* = 0.002), and the TACT measured via PA-TDI was 169 and 143 ms, respectively (*p* = 0.001).

**Table 1:** Follow-up stroke

	Patients without stroke (*n* = 366)	Patients with stroke (*n* = 10)	*p* value
Sex (male)	198 (54.1%)	5 (50%)	0.79
Age, years	61 (IQR, 22)	75.4 ± 15.4	0.003
Comorbidities			
Systolic blood pressure, mmHg	125 (IQR 15)	132 ± 8.5	0.11
Smoker status	107 (29.2%)	3 (30%)	0.95
Coronary artery disease	114 (31.1%)	2 (20%)	0.45
Heart failure	25 (6.8%)	0 (0%)	0.39
Peripheral artery disease	40 (10.9%)	4 (40%)	0.005
Diabetes mellitus	56 (15.4%)	3 (30%)	0.21
COPD	13 (3.6%)	0 (0%)	0.54
Medication			
Beta-blocker	135 (36.9%)	7 (70%)	0.03
ACE inhibitor	173 (47.3%)	6 (60%)	0.42
MRA	19 (5.2%)	1 (10%)	0.5
CCB	56 (15.3%)	3 (30%)	0.2
SAPT	103 (28.1%)	3 (30%)	0.89
DAPT	39 (10.6%)	0 (0%)	0.27
OAC	16 (4.3%)	0 (0%)	0.49
Echocardiography			
Left atrium, mm	36 (IQR, 7)	36 (IQR, 4)	0.98
Ejection fraction, %	61 (IQR, 7)	59 (IQR, 7)	0.40
PA-TDI [ms]	143.02 (IQR, 23)	163.13 (IQR 33)	< 0.001
CHA_2_DS_2_-VASc score	2 (IQR, 2)	3.5 (IQR, 2)	0.016

*IQR* interquartile range, *COPD* chronic obstructive pulmonary disease, *ACE* Angiotensin converting enzyme, *MRA* mineralocorticoid receptor antagonist, *CCB* calcium channel blocker, *NYHA* New York Heart Association, *SAPT* single antiplatelet therapy (i.e., Aspirin or Clopidogrel), *DAPT* dual antiplatelet therapy (Aspirin and Clopidogrel, Aspirin and Ticagrelor or Aspirin and Prasugrel), *OAC* oral anticoagulation therapy (Warfarin, Dabigatran or Rivaroxaban), *PA-TDI* P wave to A’ wave in tissue Doppler imaging

The clinical characteristics of the patients with newly diagnosed AF (*n* = 22) during follow-up and those without AF (*n* = 354) are presented in Table 2. Patients with AF were older and suffered significantly more frequently from diabetes mellitus and coronary artery disease, which is reflected by a higher virtual CHA_2_DS_2_-VASc score.

**Table 2:** Follow-up atrial fibrillation

	Patients without atrial fibrillation (*n* = 354)	Patients with atrial fibrillation (*n* = 22)	*p* value
Sex (male)	190 (53.7%)	13 (59.1%)	0.48
Age, years	61 (IQR, 23)	75.5 (IQR, 16)	0.001
Comorbidities			
Systolic blood pressure, mmHg	125 (IQR, 15)	125.5 (IQR, 21)	0.6
Smoker status	105 (29.7%)	5 (22.7%)	0.48
Coronary artery disease	105 (29.7%)	11 (50%)	0.04
Heart failure	23 (6.5%)	2 (9.1%)	0.63
Peripheral artery disease	41 (11.6%)	3 (13.6%)	0.77
Diabetes mellitus	50 (14.2%)	9 (40.9%)	0.001
COPD	12 (3.4%)	1 (4.5%)	0.77
Medication			
Beta-blocker	125 (35.3%)	17 (77.3%)	0.001
ACE inhibitor	159 (44.9%)	20 (90.9%)	0.001
MRA	17 (4.8%)	3 (13.6%)	0.07
CCB	55 (15.5%)	4 (18.2%)	0.74
SAPT	101 (28.5%)	5 (22.7%)	0.55
DAPT	38 (10.7%)	1 (4.5%)	0.35
OAC	14 (3.9%)	2 (9%)	0.24
Echocardiography			
Left atrium, mm	36 (IQR, 6)	36.7 ± 5.1	0.1
Ejection fraction, %	61 (IQR, 7)	60 (IQR, 7)	0.93
PA-TDI [ms]	143 (IQR 23)	161.4 (IQR 19)	< 0.001
CHA_2_DS_2_-VASc score	2 (IQR, 2)	4 (IQR, 1)	0.001

*IQR* interquartile range, *COPD* chronic obstructive pulmonary disease, *ACE* Angiotensin converting enzyme, *MRA* mineralocorticoid receptor antagonist, *CCB* calcium channel blocker, *NYHA* New York Heart Association, *SAPT* single antiplatelet therapy (i.e., Aspirin or Clopidogrel), *DAPT* dual antiplatelet therapy (Aspirin and Clopidogrel, Aspirin and Ticagrelor or Aspirin and Prasugrel), *OAC* oral anticoagulation therapy (Warfarin, Dabigatran or Rivaroxaban), *PA-TDI* P wave to A’ wave in tissue Doppler imaging

### The predictors of stroke

The univariate regression analysis revealed that both rFSRP and TACT as well as the CHA_2_DS_2_-VASc score were statistically significant predictors of stroke incidence (Table 3).

**Table 3:** Cox regression analysis

Variable	Univariate analysis	
	HR (95% CI)	*p* value
rFSRP, %	2.18 (1.50–3.18)	< 0.001
10 ms change in PA-TDI, ms	1.94 (1.49–2.54)	< 0.001
LA, mm	0.98 (0.87–1.11)	0.85
CHA_2_DS_2_-VASc	1.69 (1.09–2.62)	0.02

*HR* hazard ratio, *CI* confidence interval, *rFSRP* revised Framingham stroke risk profile, *PA-TDI* P wave to A’ wave in tissue Doppler imaging, *LA* left atrium

The relationship between the number of patients with primary/secondary outcomes and the TACT in ms is shown in Online Resource 2 (panel B). After exclusion of patients with newly diagnosed AF, the regression analysis again yielded a significant result (Table 4). Interestingly, the CHA_2_DS_2_-VASc score failed to reach statistical significance after excluding these patients.

**Table 4:** Cox regression analysis after excluding newly diagnosed patients with atrial fibrillation

Variable	Univariate analysis	
	HR (95% CI)	*p* value
rFSRP, %	1.94 (1.20–3.14)	0.006
10 ms change in PA-TDI, ms	1.85 (1.34–2.59)	< 0.001
CHA_2_DS_2_-VASc	1.57 (0.97–2.52)	0.06

*HR* hazard ratio, *CI* confidence interval, *rFSRP* revised Framingham stroke risk profile, *PA-TDI* P wave to A’ wave in tissue Doppler imaging, *LA* left atrium

### The predictive values of TACT for stroke

**Fig. 1:**
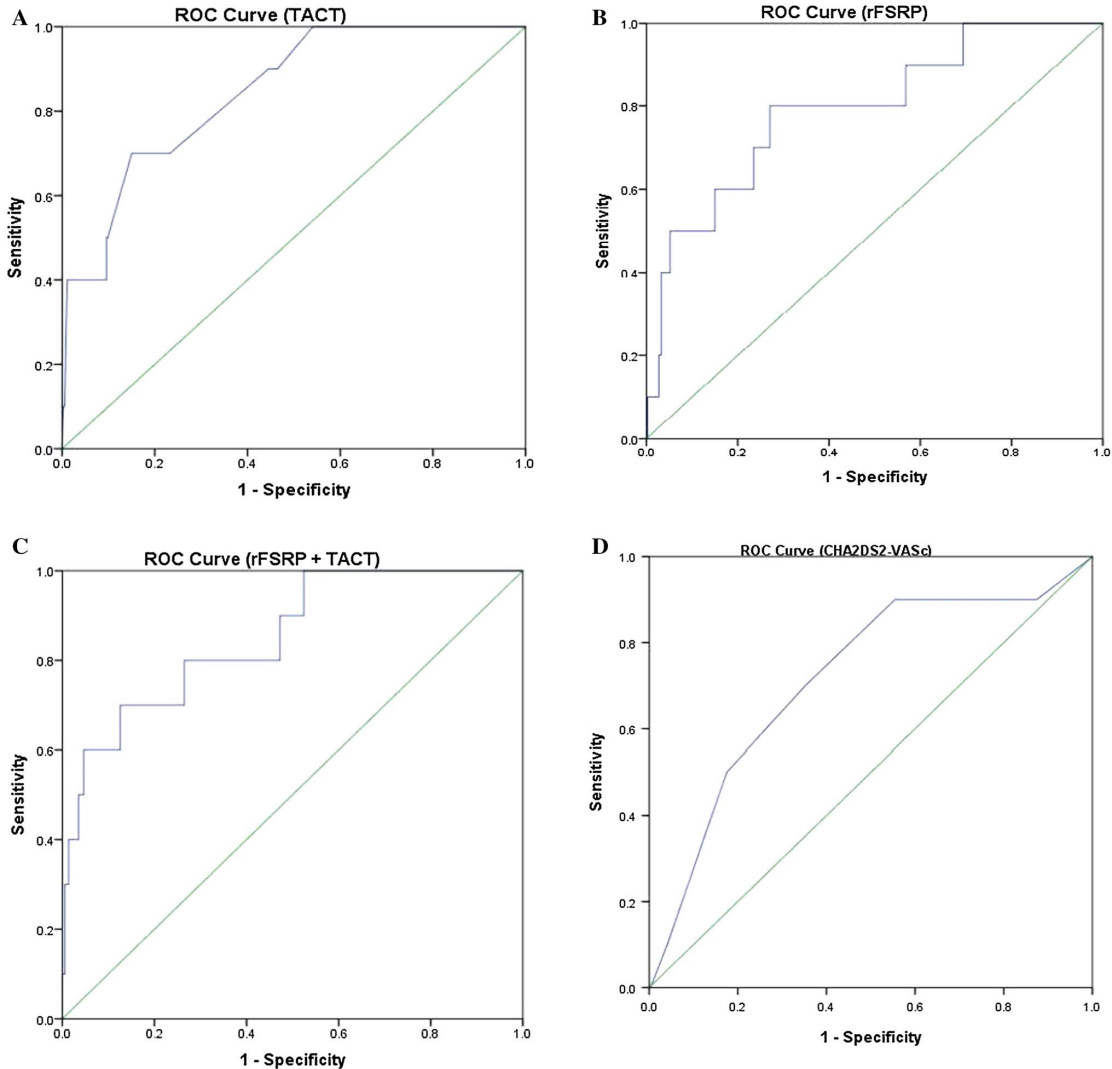
ROC analysis for TACT (panel **A**), rFSRP (panel **B**), rFSRP + TACT (panel **C**), CHA_2_DS_2_VASc score (panel **D**) *ROC* receiver operating characteristic, *TACT* total atrial conduction time, *rFSRP* revised Framingham stroke risk profile

The ROC curve analysis is shown in Fig. 1 (panels A–D). Accordingly, the rFSRP and TACT had an area under the curve (AUC) of 0.79 and 0.84, respectively, whereas the combination of both variables showed an AUC of 0.85 for subsequent stroke incidence. The CHA_2_DS_2_-VASc score showed a modest AUC of 0.71 for subsequent stroke incidence.

A complete analysis of the specificity, sensitivity, positive predictive value and negative predictive value as well as the accuracy for the TACT, rFSRP and the combination of the two (using the appropriate cut-offs) is presented in Table 5.

**Table 5:** Sensitivity, specificity, positive predictive value, negative predictive value and accuracy for the studied predictors

Variable	Sensitivity [%]	Specificity [%]	Positive predictive value [%]	Negative predictive value [%]	Accuracy [%]
TACT	70	85	11.3	99	84.5
rFSRP	80	73	7.4	99.2	73.1
TACT + rFSRP	70	87.4	13.2	99	87

*rFSRP* revised Framingham stroke risk profile, *TACT* total atrial conduction time

### The difference in stroke incidence by TACT value

**Fig. 2:**
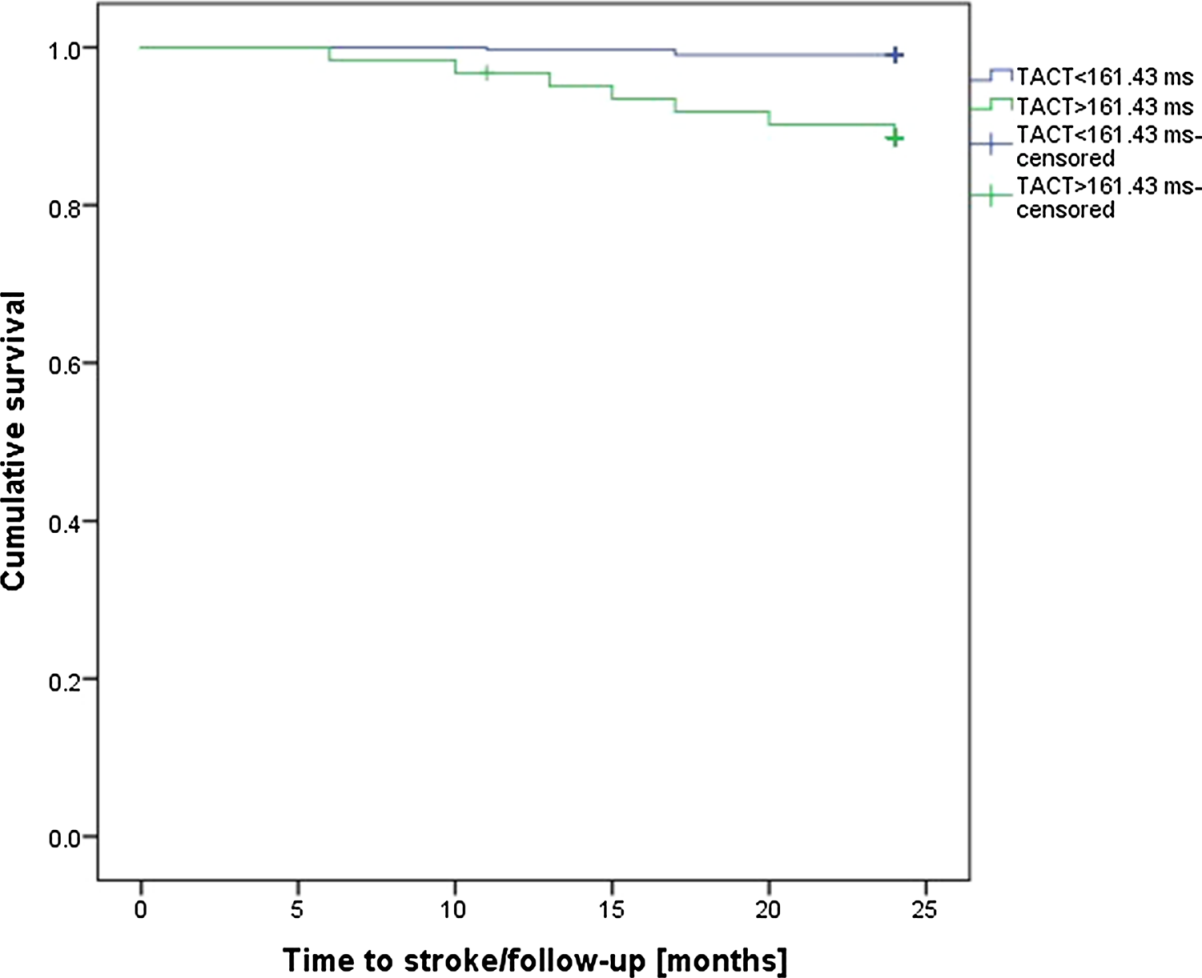
Kaplan–Meier analysis

The optimal TACT cutoff identified using the Youden index was 161.43 ms. The Kaplan–Meier curve using this cutoff demonstrated that patients with a TACT value higher than 161.43 ms had a significantly increased risk of suffering a stroke during 2 years of follow-up (log-rank Mantel–Cox, 22.35; *p* < 0.001; Fig. 2). The baseline characteristics of the cohort adjusted for this PA-TDI cutoff are presented in the Online Resource 4.

## Discussion

We investigated TACT as a potential new tool in the prediction of stroke in 376 consecutive patients in our echocardiography laboratory without clinically apparent AF. The main findings of this study are as follows:

1. Patients with a TACT higher than 161.43 ms have a significantly higher risk of stroke after 2 years despite having no prior history of AF or stroke.
2. The TACT improved the stroke prediction of the rFSRP (AUC of 0.79 for rFSRP to AUC of 0.85 for rFSRP and TACT).

TACT is a well-studied echocardiographic parameter in patients with AF. TACT has been reported to predict new-onset AF [12], stroke after catheter ablation of AF [18], postoperative AF after cardiac surgery [5], and occult AF after embolic stroke of unknown source (ESUS) [16]. Moreover, Leung et al. demonstrated in a well-powered recent study that the assessment of LA reservoir strain and PA-TDI on echocardiography after initial CHA_2_DS_2_-VASc scoring provides additional risk stratification for stroke in patients with AF [6].

Our study investigated the TACT measured noninvasively as the PA-TDI interval to predict the occurrence of stroke in patients without known AF or stroke. To our knowledge, this is the first study to investigate TACT in this subset of patients. The TACT further supports the existence of atrial cardiopathy as a substrate for thromboembolisms even in the absence of AF.

Currently, there is no clear definition of atrial cardiopathy in the literature. This concept is a complex interplay of electric, echocardiographic, and biochemical abnormalities. The ARCADIA trial [10] is currently assessing whether apixaban is superior to aspirin for the prevention of recurrent stroke in patients with ESUS and atrial cardiopathy. In this trial, atrial cardiopathy was defined as a P-wave terminal force of > 5000 μV*ms in ECG lead V1, serum NT-proBNP of > 250 pg/mL, and LA diameter index of ≥ 3 cm/m^2^ on echocardiogram. However, this definition does not include any echocardiographic markers correlated with atrial fibrosis, including TACT, LA reservoir strain, and mitral regurgitation.

The TACT cutoff for stroke (161.43 ms) in our model was higher than that for AF (145 ms) after ESUS [16]. Moreover, only 2 of the 10 patients with stroke had AF diagnosed during follow-up. This result suggests that ischemic stroke in patients with prolonged TACT was not necessarily caused by AF alone. The prolongation of TACT may reflect atrial cardiopathy, which has been reported to be a possible cause of both AF and ischemic stroke.

In this study, the LA diameter was not associated with the risk of subsequent stroke incidence (*p* = 0.85 in the univariate Cox regression analysis). In our cohort, as shown in Online Resource 3, most of the enrolled patients did not have coronary or peripheral artery disease. Perhaps because of this, the LA diameter was almost normal. The LA diameter and TACT in our study showed a statistically significant correlation. However, this was a very small positive correlation (correlation coefficient = 0.34, as shown in Online Resource 5). The key differences between our cohort and the patient population in the ARCADIA trial are age ≥ 45 years and history of ESUS, both of which increase the pretest probability of having a dilated LA.

In patients with ESUS, both dabigatran and rivaroxaban failed to show superiority over aspirin in preventing recurrent strokes [19, 20]. The choice of anticoagulant for patients with ESUS seems to represent an unbalanced risk–benefit ratio. Theoretically, patients with subclinical AF and ESUS should be anticoagulated. In this context, our data suggest that the TACT is used as a tool to improve risk stratification in patients without AF in need of anticoagulation.

This hypothesis should be further confirmed in larger prospective studies with continuous rhythm monitoring to evaluate the benefit of TACT as a possible risk index.

Our study has several limitations. First, this was a single-center, prospective cohort study. In addition, rhythm monitoring was routinely conducted through 12-lead and Holter ECGs, but the secondary endpoint (AF detection) may have missed asymptomatic paroxysmal AF. A further limitation is that we performed a sample size calculation with one-tailed testing under the alternate hypothesis that patients with prolonged TACT have a higher stroke incidence than those without. A detailed inter- and intra-observer analysis was not possible, however, the PA-TDI was measured by experienced cardiologists certified in adult transthoracic echocardiography by the European Association of Cardiovascular Imaging.

Taking into account the low number of patients with stroke and atrial fibrillation during the follow-up period, we refrained from performing a multivariate regression analysis, as the multivariate model was underpowered.

Finally, patients with more comorbidities may be underrepresented because of the enrollment criteria.

### Conclusion

This study showed PA-TDI improves the predictive power when used in addition to the rFSRP. Our findings support that of atrial cardiopathy, and the PA-TDI may represent an important tool to risk stratify patients who may need closer rhythm monitoring and distinct indications for anticoagulation. However, these findings must be evaluated in larger cohorts.

## Supplementary Information

Below is the link to the electronic supplementary material.

Supplementary file1 (PDF 201 KB)

## References

[1] Rajsic S, Gothe H, Borba HH, Sroczynski G, Vujicic J, Toell T, Siebert U (2019). Economic burden of stroke: a systematic review on post-stroke care. Eur J Health Econ.

[2] Luengo-Fernandez R, Violato M, Candio P, Leal J (2020). Economic burden of stroke across Europe: a population-based cost analysis. Eur Stroke J.

[3] Kamel H, Okin PM, Elkind MS, Iadecola C (2016). Atrial fibrillation and mechanisms of stroke: time for a new model. Stroke.

[4] Wolf PA, Abbott RD, Kannel WB (1991). Atrial fibrillation as an independent risk factor for stroke: the Framingham Study. Stroke.

[5] Müller P, Deneke T, Schiedat F, Bösche L, Strauch J, Dietrich JW, Vogt M, Tannapfel A, Stiegler H, Mügge A, Ewers A (2013). Increased preoperative serum apoptosis marker fas ligand correlates with histopathology and new-onset of atrial fibrillation in patients after cardiac surgery. J Cardiovasc Electrophysiol.

[6] Leung M, van Rosendael PJ, Abou R, Ajmone Marsan N, Leung DY, Delgado V, Bax JJ (2018). Left atrial function to identify patients with atrial fibrillation at high risk of stroke: new insights from a large registry. Eur Heart J.

[7] Dufouil C, Beiser A, McLure LA, Wolf PA, Tzourio C, Howard VJ, Westwood AJ, Himali JJ, Sullivan L, Aparicio HJ, Kelly-Hayes M, Ritchie K, Kase CS, Pikula A, Romero JR, D’Agostino RB, Samieri C, Vasan RS, Chêne G, Howard G, Seshadri S (2017). Revised Framingham stroke risk profile to reflect temporal trends. Circulation.

[8] Jordan K, Yaghi S, Poppas A, Chang AD, Mac Grory B, Cutting S, Burton T, Jayaraman M, Tsivgoulis G, Sabeh MK, Merkler AE, Kamel H, Elkind MS, Furie K, Song C (2019). Left atrial volume index is associated with cardioembolic stroke and atrial fibrillation detection after embolic stroke of undetermined source. Stroke.

[9] Kamel H, Hunter M, Moon YP, Yaghi S, Cheung K, Di Tullio MR, Okin PM, Sacco RL, Soliman EZ, Elkind MS (2015). Electrocardiographic left atrial abnormality and risk of stroke: northern manhattan study. Stroke.

[10] Kamel H, Longstreth WT, Tirschwell DL, Kronmal RA, Broderick JP, Palesch YY, Meinzer C, Dillon C, Ewing I, Spilker JA, Di Tullio MR, Hod EA, Soliman EZ, Chaturvedi S, Moy CS, Janis S, Elkind MS (2019). The AtRial cardiopathy and antithrombotic drugs in prevention after cryptogenic stroke (ARCADIA) randomized trial: rationale and methods. Int J Stroke.

[11] Lang RM, Badano LP, Mor-Avi V, Afilalo J, Armstrong A, Ernande L, Flachskampf FA, Foster E, Goldstein SA, Kuznetsova T, Lancellotti P, Muraru D, Picard MH, Rietzschel ER, Rudski L, Spencer KT, Tsang W, Voigt J-U (2015). Recommendations for cardiac chamber quantification by echocardiography in adults: an update from the American society of echocardiography and the European association of cardiovascular imaging. J Am Soc Echocardiogr.

[12] Vos CBD, Weijs B, Crijns HJGM, Cheriex EC, Palmans A, Habets J, Prins MH, Pisters R, Nieuwlaat R, Tieleman RG (2009). Atrial tissue Doppler imaging for prediction of new-onset atrial fibrillation. Heart.

[13] Fischer U, Baumgartner A, Arnold M, Nedeltchev K, Gralla J, De Marchis GM, Kappeler L, Mono ML, Brekenfeld C, Schroth G, Mattle HP (2010). What is a minor stroke?. Stroke.

[14] Sacco RL, Kasner SE, Broderick JP, Caplan LR, Connors JJ, Culebras A, Elkind MS, George MG, Hamdan AD, Higashida RT, Hoh BL, Janis LS, Kase CS, Kleindorfer DO, Lee J-M, Moseley ME, Peterson ED, Turan TN, Valderrama AL, Vinters HV (2013). An updated definition of stroke for the 21st century. Stroke.

[15] Asplund K, Karvanen J, Giampaoli S, Jousilahti P, Niemelä M, Broda G, Cesana G, Dallongeville J, Ducimetriere P, Evans A, Ferrières J, Haas B, Jorgensen T, Tamosiunas A, Vanuzzo D, Wiklund P-G, Yarnell J, Kuulasmaa K, Kulathinal S (2009). Relative risks for stroke by age, sex, and population based on follow-up of 18 European populations in the MORGAM project. Stroke.

[16] Müller P, Ivanov V, Kara K, Klein-Wiele O, Forkmann M, Piorkowski C, Blockhaus C, Dimitroulis D, Afzal S, Shin DI, Kelm M, Makimoto H, Mügge A (2017). Total atrial conduction time to predict occult atrial fibrillation after cryptogenic stroke. Clin Res Cardiol.

[17] Wang MC, Li S (2013). ROC Analysis for multiple markers with tree-based classification. Lifetime Data Anal.

[18] den Uijl DW, Gawrysiak M, Tops LF, Trines SA, Zeppenfeld K, Schalij MJ, Bax JJ, Delgado V (2011). Prognostic value of total atrial conduction time estimated with tissue Doppler imaging to predict the recurrence of atrial fibrillation after radiofrequency catheter ablation. Europace.

[19] Diener HC, Sacco RL, Easton JD, Granger CB, Bernstein RA, Uchiyama S, Kreuzer J, Cronin L, Cotton D, Grauer C, Brueckmann M, Chernyatina M, Donnan G, Ferro JM, Grond M, Kallmünzer B, Krupinski J, Lee BC, Lemmens R, Masjuan J, Odinak M, Saver JL, Schellinger PD, Toni D, Toyoda K (2019). Dabigatran for prevention of stroke after embolic stroke of undetermined source. N Engl J Med.

[20] Hart RG, Sharma M, Mundl H, Kasner SE, Bangdiwala SI, Berkowitz SD, Swaminathan B, Lavados P, Wang Y, Wang Y, Davalos A, Shamalov N, Mikulik R, Cunha L, Lindgren A, Arauz A, Lang W, Czlonkowska A, Eckstein J, Gagliardi RJ, Amarenco P, Ameriso SF, Tatlisumak T, Veltkamp R, Hankey GJ, Toni D, Bereczki D, Uchiyama S, Ntaios G, Yoon B-W, Brouns R, Endres M, Muir KW, Bornstein N, Ozturk S, O’Donnell MJ, De Vries Basson MM, Pare G, Pater C, Kirsch B, Sheridan P, Peters G, Weitz JI, Peacock WF, Shoamanesh A, Benavente OR, Joyner C, Themeles E, Connolly SJ (2018). Rivaroxaban for Stroke Prevention after Embolic Stroke of Undetermined Source. N Engl J Med.

