# Supplementary material for: Total atrial conduction time provides novel information in prediction for stroke in patients with sinus rhythm
Source: Heart Vessels. 2022 Oct 20;38(4):543–50. doi: 10.1007/s00380-022-02189-7 (PMC9986205; doi:10.1007/s00380-022-02189-7)
Supplement: Supplementary file 1 — Supplementary file1 (PDF 201 KB) [file 380_2022_2189_MOESM1_ESM.pdf]

Article title:

Total atrial conduction time provides novel information in prediction for stroke in patients with sinus rhythm

Authors:

Bejinariu, AG[a], MD, Schilling, M[a], MD, Müller, P[a], MD, Clasen, L[a], MD, Gerguri, S[a], MD, Angendohr, S[a], MD, Katsianos, S[a], Schmidt, J[a], MD, Brinkmeyer, C[a], MD, Meuth, SG[b], MD, PhD, Kelm, M[a,c], MD, PhD, Makimoto, H[a], MD, PhD

From the [a]Division of Cardiology, Pulmonology and Vascular Medicine, Medical Faculty, Heinrich-Heine University, Moorenstrasse 5, 40225 Düsseldorf, Germany, [b]Division of Neurology, Medical Faculty, Heinrich-Heine University, Moorenstrasse 5, 40225 Düsseldorf, Germany, [c]CARID, Cardiovascular Research Institute Düsseldorf, Medical Faculty, Heinrich-Heine-University, Moorenstrasse 5, 40225 Düsseldorf, Germany

Journal name:

Heart and Vessels

Corresponding author and e-mail address

alexandru-gabriel.bejinariu@med.uni-duesseldorf.de

Online Resource 1. Measurement of the PA-TDI interval

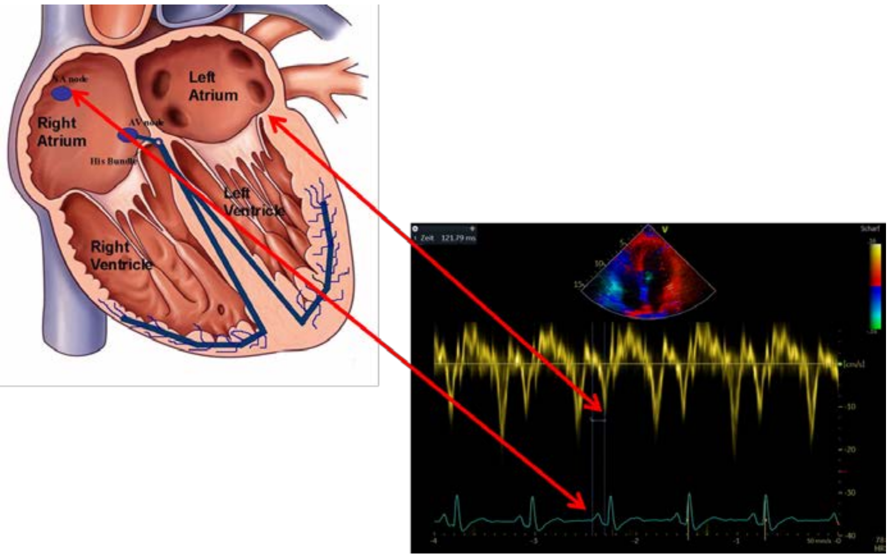

Abbreviation: PA-TDI = P wave to A’ wave in tissue doppler imaging

Online Resource 2. Panel A Patient's selection and follow-up. **Panel B** Number of patients meeting the outcome in relationship with the TACT

A

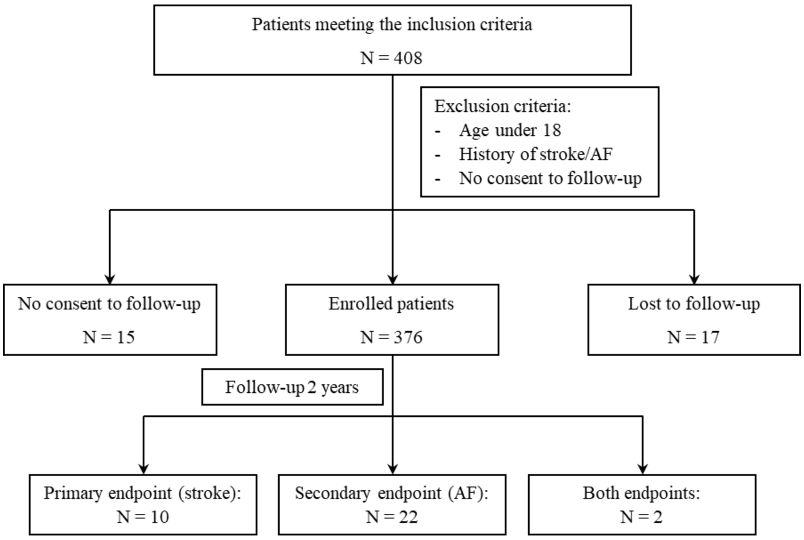

B

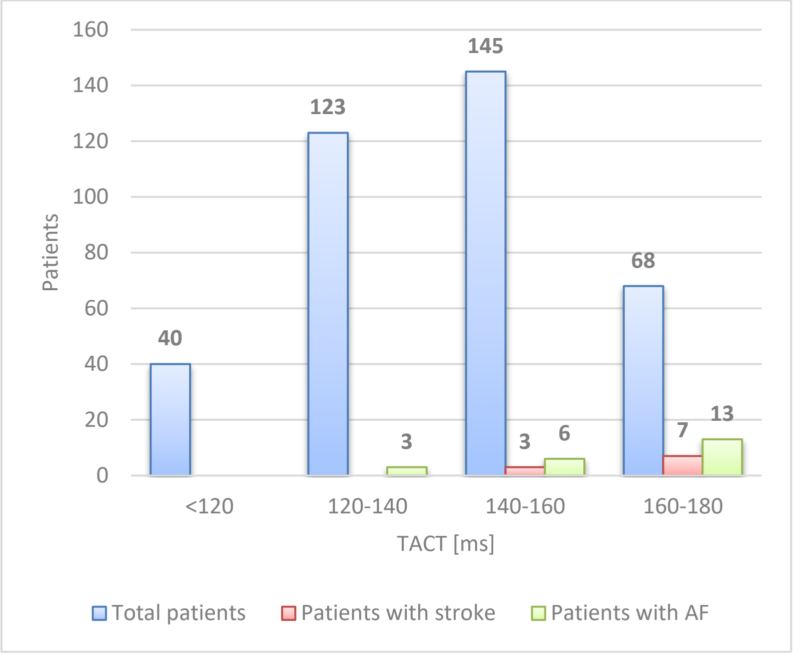

Abbreviation: AF = atrial fibrillation, TACT = total atrial conduction time

Online Resource 3. Baseline characteristics of all patients

**Sex (male)**	203 (54%)
**Age, years**	61 (IQR, 23)
**Comorbidities**	
Hypertension	224 (59.6%)
Smoker status	110 (29.3%)
Coronary artery disease	116 (30.9%)
Heart failure	25 (6.6%)
Peripheral artery disease	44 (11.7%)
Diabetes mellitus	59 (15.7%)
COPD	13 (3.5%)
**Medication**	
Beta-blocker	142 (37.8%)
ACE inhibitor	179 (47.6%)
MRA	20 (5.3%)
CCB	59 (15.7%)
SAPT	106 (28.1%)
DAPT	39 (10.3%)
OAC	16 (4.2%)
**Echocardiography**	
Left atrium, mm	36 (IQR, 6)
Ejection fraction, %	61 (IQR, 7)
PA-TDI [ms]	143±17
**NYHA functional class**	
Class I	225

Class II	141
Class III	10
Class IV	0
**CHA_2DS_2-VASc score**	2 (IQR, 2)

Abbreviation: IQR = interquartile range, COPD = chronic obstructive pulmonary disease, ACE = Angiotensin converting enzyme, MRA = mineralocorticoid receptor antagonist, CCB = calcium channel blocker, NYHA = New York Heart Association, SAPT = single antiplatelet therapy (i.e., Aspirin or Clopidogrel), DAPT = dual antiplatelet therapy (Aspirin and Clopidogrel, Aspirin and Ticagrelor or Aspirin and Prasugrel), OAC = oral anticoagulation therapy (Warfarin, Dabigatran or Rivaroxaban), PA-TDI = P wave to A' wave in tissue doppler imaging

Online Resource 4. Online Resource 4 – Baseline characteristics adjusted for the PA-TDI cut off

	Patients with PA-TDI < 161.43 ms (n = 314)	Patients with PA-TDI ≥ 161.43 ms (n = 62)	p value
**Sex (male)**	167 (53.2%)	36 (58.1%)	0.48
**Age, years**	58 (IQR, 21)	74 (IQR, 11)	0.001
**Comorbidities**			
Systolic blood pressure, mmHg	125 (IQR, 15)	127.5 (IQR, 16)	0.72
Smoker status	92 (29.3%)	18 (29%)	0.96
Coronary artery disease	82 (26.1%)	34 (54.8%)	0.001
Heart failure	20 (6.4%)	5 (8.1%)	0.62
Peripheral artery disease	25 (8%)	19 (30.6%)	0.001
Diabetes mellitus	40 (12.8%)	19 (30.6%)	0.001
COPD	10 (3.2%)	3 (4.8%)	0.51
**Medication**			
Beta-blocker	103 (32.8%)	39 (62.9%)	0.001
ACE inhibitor	131 (41.7%)	48 (77.4%)	0.001
MRA	14 (4.5%)	6 (3.3%)	0.09
CCB	40 (12.7%)	19 (30.6%)	0.001
**Echocardiography**			
Left atrium, mm	35 (IQR, 6)	38 (IQR, 5)	0.001
Ejection fraction, %	61 (IQR, 7)	59.5 (IQR, 8)	0.054
**CHA_2DS_2-VASc score**	1 (IQR, 2)	3 (IQR, 1)	0.001

Abbreviation: PA-TDI = P wave to A' wave in tissue doppler imaging, IQR = interquartile range, COPD = chronic obstructive pulmonary disease, ACE = Angiotensin converting enzyme, MRA = mineralocorticoid receptor antagonist, CCB = calcium channel blocker,

Online Resource 5. Diagram showing the correlation between LA diameter and TACT

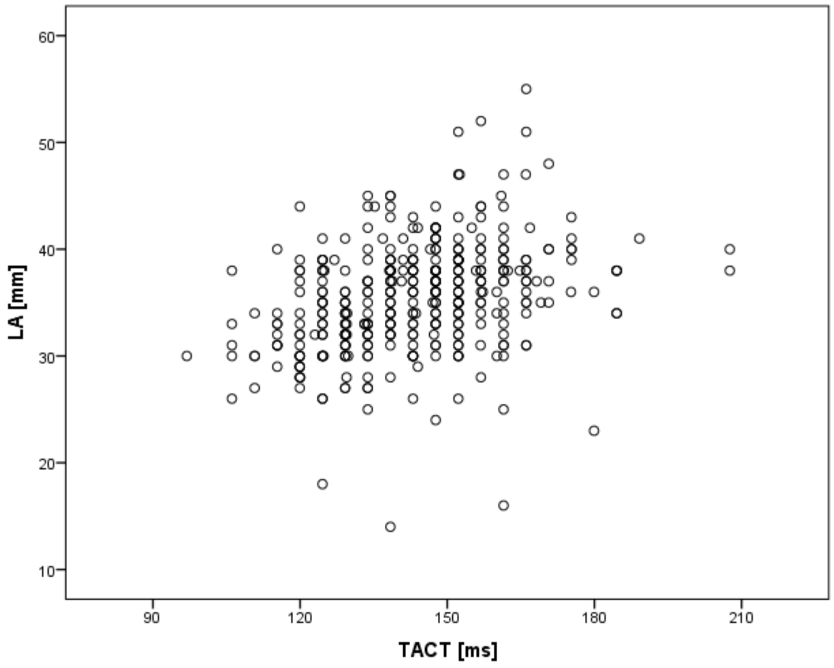

Abbreviation: LA=left atrium, TACT=total atrial conduction time
